# SEZ6L2 Is an Important Regulator of Drug-Resistant Cells and Tumor Spheroid Cells in Lung Adenocarcinoma

**DOI:** 10.3390/biomedicines8110500

**Published:** 2020-11-13

**Authors:** Jang-Seok Lee, Hee Yeon Kim, Bomyi Won, Sang Won Kang, Yong-Nyun Kim, Hyonchol Jang

**Affiliations:** 1 Research Institute, National Cancer Center, Goyang 10408, Korea; 75509@ncc.re.kr (J.-S.L.); 74790@ncc.re.kr (H.Y.K.); dhhb4@naver.com (B.W.); ynk@ncc.re.kr (Y.-N.K.); 2 Department of Life Science, Ewha Womans University, Seoul 03760, Korea; kangsw@ewha.ac.kr; 3 Department of Cancer Biomedical Science, National Cancer Center Graduate School of Cancer Science and Policy, Goyang 10408, Korea

**Keywords:** lung adenocarcinoma, distant recurrence, drug resistance, circulating tumor cell, tumor spheroid, SEZ6L2

## Abstract

Many lung cancer deaths result from relapses in distant organs, such as the brain or bones, after standard chemotherapy. For cancer cells to spread to other organs, they must survive as circulating tumor cells (CTCs) in blood vessels. Thus, reducing distant recurrence after chemotherapy requires simultaneously inhibiting drug resistance and CTC survival. Here, we investigated the molecular pathways and genes that are commonly altered in drug-resistant lung cancer cells and lung tumor spheroid (TS) cells. First, RNA sequencing was performed in drug-resistant cells and TS cells originating from H460 and A549 lung cancer cells. Bioinformatic pathway analysis showed that cell cycle-related pathways were downregulated in drug-resistant cells, and cholesterol biosynthesis-related pathways were upregulated in TS cells. Seizure-related 6 homolog-like 2 (*SEZ6L2*) was selected as a gene that was commonly upregulated in both drug-resistant cells and TS cells, and that showed elevated expression in samples from lung adenocarcinoma patients. Second, the protein expression of SEZ6L2 was analyzed by flow cytometry. The proportions of SEZ6L2 positive cells among both drug-resistant cells and TS cells was increased. Finally, as SEZ6L2 is a transmembrane protein with an extracellular region, the function of SEZ6L2 was disrupted by treatment with an anti-SEZ6L2 antibody. Treatment with the anti-SEZ6L2 antibody reduced drug resistance and TS formation. Overall, our data showed that SEZ6L2 plays an important role in drug resistance and TS formation and may be a therapeutic target for reducing distant recurrence of lung adenocarcinoma.

## 1. Introduction

Lung cancer is the leading cause of cancer deaths worldwide [1]. Overall, 85% of lung cancer patients are diagnosed with non-small cell lung cancer (NSCLC), and the two major NSCLC subtypes are lung adenocarcinoma (LUAD) and lung squamous cell carcinoma [2,3]. To date, many treatments have been developed for lung cancer, including surgery, chemotherapy, and radiation therapy. However, despite improvements in lung cancer treatment, many patients with NSCLC experience recurrence after surgery or chemotherapy [4,5,6]. Post-recurrence survival is low, and recurrence is associated with a poor prognosis [7,8]. Approximately 65% of NSCLC patients will develop persistent disease or distant metastasis to the brain or bone after surgery and postoperative adjuvant therapy [7,9,10].

The present study aimed to explore strategies to prevent tumor relapse in distant organs during and after chemotherapy. After drug treatment, only drug-resistant cancer cells are viable and can metastasize to other distant sites through blood flow. In the bloodstream, cancer cells must overcome the harsh environment to survive as circulating tumor cells (CTCs) [11]. The most important factor in the survival of CTCs is the survival in the suspended state, and tumor spheroids (TSs) are widely used as in vitro models to study cell survival in the suspended state [12,13,14]. Because molecular changes are a major factor leading to distant metastasis and resistance to lung cancer treatment [15,16,17], we investigated the common molecular features of both drug resistance and metastasis by RNA sequencing. To analyze drug resistance, we used the survived cells after treatment with three drugs (cisplatin, paclitaxel, and doxorubicin), and to analyze metastasis, we cultured TS cells in the H460 and A549 cell lines. We analyzed the RNA sequencing data and investigated gene expression profiles common to both drug resistance and metastasis.

Based on the RNA sequencing results, we focused on seizure-related 6 homolog-like 2 (*SEZ6L2*), which is a type 1 transmembrane protein. *SEZ6L2* belongs to the SEZ6 family, which is composed of *SEZ6*, *SEZ6L*, and *SEZ6L2* [18] and is upregulated in various cancers compared to the matched normal samples according to GEPIA2 (Gene Expression Profiling Interactive Analysis) webserver (http://gepia2.cancer-pku.cn/). Moreover, *SEZ6L2* has been reported as a prognostic marker for hepatocellular carcinoma [18] and found to have increased expression in ovarian cancer [19]. Previous research on lung cancer showed shorter survival times in patients with tumors exhibiting high *SEZ6L2* expression compared with no *SEZ6L2* expression, indicating that *SEZ6L2* may be a novel prognostic marker for lung cancer [20]. However, the roles of *SEZ6L2* in drug resistance and metastasis in lung cancer remain unclear.

In this study, we confirmed that *SEZ6L2* is upregulated in both drug-resistant cells and TS cells. Furthermore, we showed that inhibition of *SEZ6L2* via treatment with an anti-SEZ6L2 antibody reduced drug resistance and TS formation, suggesting that anti-SEZ6L2 antibody therapy may be an option for reducing tumor relapse after chemotherapy in LUAD.

## 2. Experimental Section

### 2.1. Cell Culture and Reagents

The human LUAD cell lines H460 and A549 were purchased from the Korean Cell Line Bank. H460 and A549 cells were cultured in RPMI medium (#SH30027.01; HyClone, Logan, UT, USA) supplemented with 10% heat-inactivated fetal bovine serum (#SH30084.03; HyClone) and 1% penicillin/streptomycin (#15140-122; Invitrogen, San Diego, CA, USA) at 37 °C in humidified incubators containing 5% CO_2_. Cell lines were authenticated and regularly checked for *Mycoplasma* at the Genomics Core Facility (National Cancer Center, Gyeonggi-do, South Korea), as described previously [21].

TS cells were cultured according to the ex vivo CTC culture method described previously [22] with some modifications. Briefly, H460 and A549 cells were cultured in TS culture medium on plates coated with poly (2-hydroxyethyl methacrylate) (#P3932; Sigma–Aldrich, St. Louis, MO, USA) at 37 °C in a humidified incubator with 5% CO_2_. The TS culture medium consisted of RPMI medium supplemented with 1× B27 (#17504-044; Invitrogen), 20 ng/mL basic fibroblast growth factor (#100-18B; PEPROTECH, Cranbury, NJ, USA), 20 ng/mL epidermal growth factor (#E9644; Sigma–Aldrich), 1% penicillin/streptomycin, and Cellmaxin plus (#C3319-020; GenDEPOT, Austin, TX, USA). TS cells were passaged at least three times for stabilization.

For drug treatment, cisplatin (#C2210000) was purchased from Sigma–Aldrich, paclitaxel (#1097) from TOCRIS (Bristol, UK), and doxorubicin (#S1208) from Selleckchem (Houston, TX, USA). H460 and A549 cells were plated, and drug treatments were added the next day. After 3 days of drug treatment, culture media were exchanged with complete fresh media. After 3 additional days, whole cells were re-plated into another dish.

The anti-SEZ6L2 antibody (#PA5-24862) was purchased from Invitrogen, normal rabbit IgG (#12-370) was purchased from EMD Millipore (Billerica, MA, USA), and goat anti-rabbit IgG (H+L) Cross-Adsorbed Secondary Antibody Alexa Fluor 488 (#A-11008) was purchased from Invitrogen.

### 2.2. RNA Sequencing and Data Analysis

RNA sequencing was performed according to a method described previously [23,24]. Preparation of the RNA library and sequencing were performed using HiSeq 2000 and HiSeq 2500 sequencing systems (Illumina, San Diego, CA, USA) by Macrogen (Seoul, Korea). The RNA sequencing data were deposited in the Gene Expression Omnibus (GEO) database under accession number GSE158638 and GSE158640. RNA sequencing data were analyzed by core analysis using ingenuity pathway analysis (IPA; QIAGEN, Redwood City, CA, USA). Differentially expressed genes (DEGs) were filtered using a fold-change expression cut-off of 2. A heatmap of the DEGs was created using MultiExperiment Viewer version 4.9.0 (mev.tm4.org). DEGs were analyzed based on canonical pathways and upstream regulators using IPA.

### 2.3. Flow Cytometry

The populations of SEZ6L2-positive cells among H460 and A549 cells were evaluated by flow cytometry using an anti-SEZ6L2 antibody. Cells were serially stained with the anti-SEZ6L2 antibody and goat anti-rabbit IgG (H+L) Cross-Adsorbed Secondary Antibody Alexa Fluor 488. Samples were analyzed at the Flow Cytometry Core Facility (National Cancer Center) using FACSVerse (BD Biosciences, San Jose, CA, USA), as described previously [25].

### 2.4. TS Formation and Antibody Treatment

Single-cell suspensions of H460 cells were plated into 96-well ultra-low attachment plates in the TS culture medium. In total, 1000 cells were plated and incubated with rabbit IgG or anti-SEZ6L2 antibody at the indicated concentration for at least 7 days. After antibody incubation, whole-cell images were obtained using the Cytation 3 cell imaging reader (BioTek, Winooski, VT, USA) and analyzed using ImageJ, as described previously [26]. TS cells with diameters of more than 10 μm were counted.

### 2.5. Statistical Analysis

Statistical analysis was performed as reported previously [21]. The data were presented as means ± standard deviation, and *p*-values were calculated using the Student’s *t*-test calculator (http://graphpad.com/quickcalcs/). All data were representative of at least three separate experiments.

## 3. Results

### 3.1. Characterization of Gene Expression Changes in Drug-Resistant Cells

**Figure 1:**
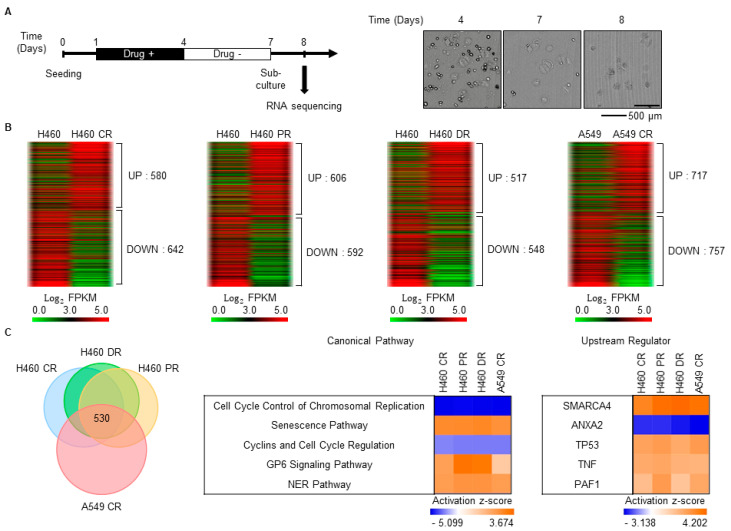
Characterization of gene expression changes in drug-resistant cells. (**A**) Schematic of the drug treatment schedule and sample preparation timeline for RNA sequencing. Representative images of H460 cells treated with paclitaxel (5 nM) were shown. (**B**) Cells that survived drug treatment according to the scheme shown in (**A**) were considered drug-resistant cells. CR: cisplatin-resistant; PR: paclitaxel-resistant; DR: doxorubicin-resistant. To generate drug-resistant H460 cells, 5 nM paclitaxel, 4 μM cisplatin, and 0.5 μM doxorubicin were applied, and 10 μM of cisplatin was applied to generate CR A549 cells. Differentially expressed genes (DEGs) between parental and drug-resistant cells were identified by RNA sequencing. DEGs with expression levels that were altered more than two-fold are shown as heatmaps produced using the Multi-Experiment Viewer. (**C**) The number of DEGs common to drug-resistant H460 and A549 cells is shown as a Venn diagram (left). These common DEGs were analyzed by core analysis using ingenuity pathway analysis (IPA). Based on the comparative analysis using IPA, the top five canonical pathways and upstream regulators were shown (middle, right).

After chemotherapy, a small number of drug-resistant cells survive, and these cells exhibit drug resistance [27]. Thus, to select drug-resistant cells, the LUAD cell line H460 was treated with cisplatin, paclitaxel, and doxorubicin, which are widely used drugs for cancer treatment. As cancer cells continue to die for a period after cessation of the anticancer drug treatment (Figure 1A, right), the culture medium was exchanged for fresh medium 3 days after treatment with the anticancer drugs, and the cells were then cultured for an additional 3 days. After re-plating, the attached cells were considered drug-resistant surviving cells (Figure 1A). Whole-cell mRNA expression levels of parental, cisplatin-resistant (CR), paclitaxel-resistant (PR), and doxorubicin-resistant (DR) H460 cells were analyzed by RNA sequencing. A total of 1000–1200 genes were differentially expressed by more than twofold between the parental and drug-resistant H460 cells (Figure 1B). DEGs between the parental and CR A549 cells reported previously [27] were also analyzed (Figure 1B). A total of 530 DEGs were common in the CR, PR, and DR H460 cells and CR A549 cells relative to the parental cells (Figure 1C, left). Canonical pathway analysis of these common DEGs by IPA showed that cell-cycle-related pathways were generally downregulated (Figure 1C, middle), which is consistent with a previous report that the proliferation of drug-resistant cells is inhibited [27]. Meanwhile, the nucleotide excision repair (NER) pathway, which is closely involved in drug resistance [28], was generally upregulated (Figure 1C, middle). Upstream regulators that control the expression of common DEGs were also analyzed using IPA. Among the upstream regulators, annexin A2 (*ANXA2*) was repressed, and switch/sucrose non-fermentable (SWI/SNF) related regulator of chromatin (*SMARCA4*, also known as *BRG1*), tumor protein 53 (*TP53*), tumor necrosis factor *(TNF*), and polymeraseassociated factor1 (*PAF1*) homolog, Paf1/RNA polymerase II complex component (*PAF1*) were activated (Figure 1C, right).

### 3.2. Characterization of Gene Expression Changes in TS Cells

**Figure 2:**
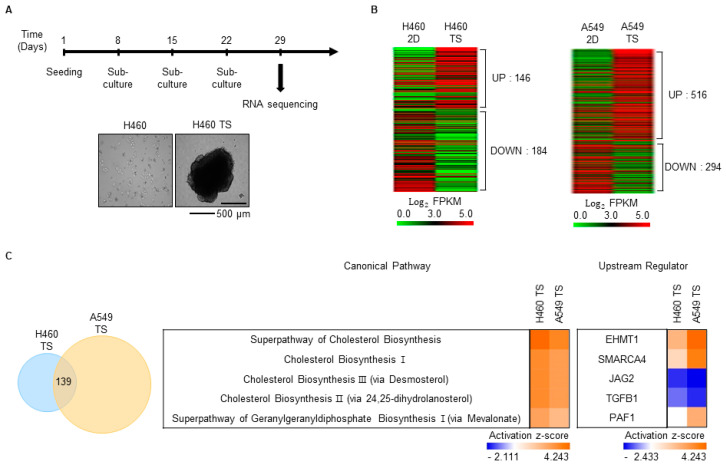
Characterization of gene expression changes in tumor spheroid (TS) cells. (**A**) Schematic of the TS cell culture schedule and sample preparation timeline for RNA sequencing. Representative images of H460 cells cultured under two-dimensional (2D) and TS cell conditions at the time of sample preparation were shown. (**B**) DEGs between 2D- and TS-cultured H460 or A549 cells were analyzed by RNA sequencing. DEGs with expression levels that were altered by more than two-fold were shown as heatmaps produced using the Multi-Experiment Viewer. (**C**) The number of DEGs common to H460 and A549 TS cells was shown in a Venn diagram (left). These common DEGs were analyzed by core analysis using IPA. Based on comparative analysis using IPA, the top five canonical pathways and upstream regulators were shown (middle, right).

For cancer cells to recur in other organs, they must be able to survive in a suspended state in blood vessels. To obtain TS cells, H460 and A549 cells were cultured according to a previously reported ex vivo CTC culture method [22] with some modification. To distinguish TS cells from cell aggregates and to stabilize them, TS cells were passaged three times prior in preparation for RNA sequencing (Figure 2A). In total, 330 and 810 genes were differentially expressed by more than twofold, respectively, in H460 and A549 TS cells compared with the corresponding cells cultured under two-dimensional (2D) conditions (Figure 2B). Of those genes, 139 were common DEGs between the 2D-cultured cells and both the H460 and A549 TS cells (Figure 2C, left). Canonical pathway analysis of these common DEGs using IPA showed that cholesterol biosynthesis-related pathways were generally upregulated (Figure 2C, middle). Upstream regulator analysis showed that euchromatic histone lysine methyltransferase 1 (EHMT1), SMARCA4, and PAF1 were activated, whereas jagged canonical Notch ligand 2 (JAG2) and transforming growth factor beta 1 (TGFB1) were repressed (Figure 2C, right).

### 3.3. SEZ6L2 Is Commonly Upregulated in Drug-Resistant Cells and TS Cells

**Figure 3:**
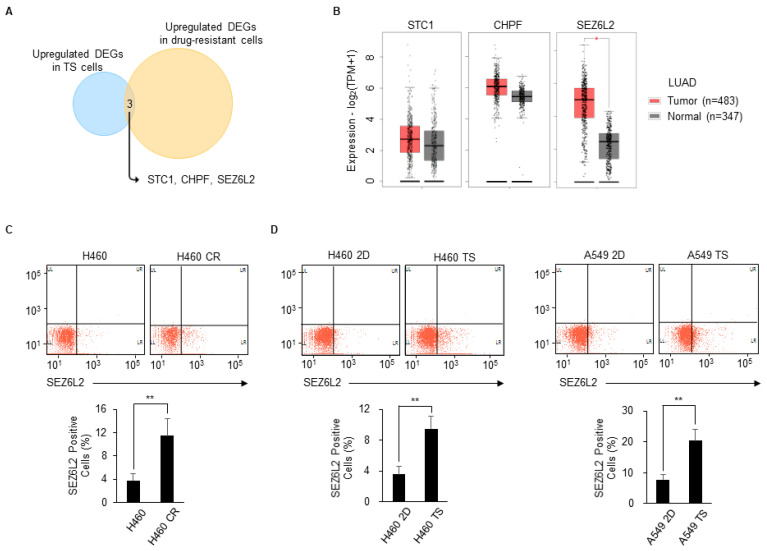
Seizure-related 6 homolog-like 2 (SEZ6L2) is commonly upregulated in drug-resistant cells and TS cells. (**A**) Commonly upregulated DEGs in drug-resistant cells and TS cells compared with the corresponding control cells were shown. Only three genes (*STC1*, *CHPF*, and *SEZ6L2*) were commonly upregulated. (**B**) The expression levels of these three genes were compared between lung adenocarcinoma (LUAD) patients and matched normal samples by the GEPIA2 webserver (http://gepia2.cancer-pku.cn/). (**C**) Expression levels of SEZ6L2 in H460 parental cells and cisplatin-resistant (CR) cells were analyzed by flow cytometry (FACSVerse) using an anti-SEZ6L2 antibody. The proportion of SEZ6L2-positive cells was expressed as the mean ± standard deviation (n = 3). ** *p* < 0.01 relative to H460 parental cells. (**D**) Expression levels of SEZ6L2 in H460 and A549 2D- and TS cells were analyzed by flow cytometry. The proportion of SEZ6L2-positive cells was expressed as the mean ± standard deviation (n = 3). ** *p* < 0.01 relative to 2D-cultured cells.

Initially, we aimed to discover common pathways and upstream regulators governing drug resistance and TS formation to identify anticancer strategies that simultaneously inhibit both processes. However, canonical pathway analysis showed no common pathways between drug-resistant cells and TS cells, while among the upstream regulators, only SMARCA4 and PAF1 were commonly activated (Figure 1C and Figure 2C). As the mRNA expression levels of *SMARCA4* and *PAF1* were not significantly altered in drug-resistant cells and TS cells compared with control cells (data not shown), activation of SMARCA4 and PAF1 appears to occur at the post-transcriptional level. Therefore, we searched for genes with increased expression in both drug-resistant cells and TS cells. Only three genes, stanniocalcin 1 (*STC1*), chondroitin polymerizing factor (*CHPF*), and seizure-related 6 homolog like 2 (*SEZ6L2*), were upregulated in all drug-resistant cells and TS cells relative to the corresponding control cells (Figure 3A). The expression levels of these three genes were compared between LUAD patients and matched normal samples by the GEPIA2 (Gene Expression Profiling Interactive Analysis) webserver. As only *SEZ6L2* was upregulated in tumors compared with normal samples (Figure 3B), we focused on *SEZ6L2*. First, we investigated whether the SEZ6L2 protein level was also increased in drug-resistant cells and TS cells. SEZ6L2 is expressed on the cell surface [18,20], and therefore flow cytometric analysis was performed using an anti-SEZ6L2 antibody. SEZ6L2 was detected in less than 4% of H460 parental cells, whereas more than 10% of CR H460 cells were positive for SEZ6L2 (Figure 3C). In addition, in both H460 and A549 cells, the population of SEZ6L2-positive cells among TS cells was increased by twofold compared with 2D cultures (Figure 3D). These results suggest that the population of SEZ6L2-positive cells increases during the development of drug resistance and TS formation.

### 3.4. Anti-SEZ6L2 Antibody Treatment Inhibits Drug Resistance and TS Formation

**Figure 4:**
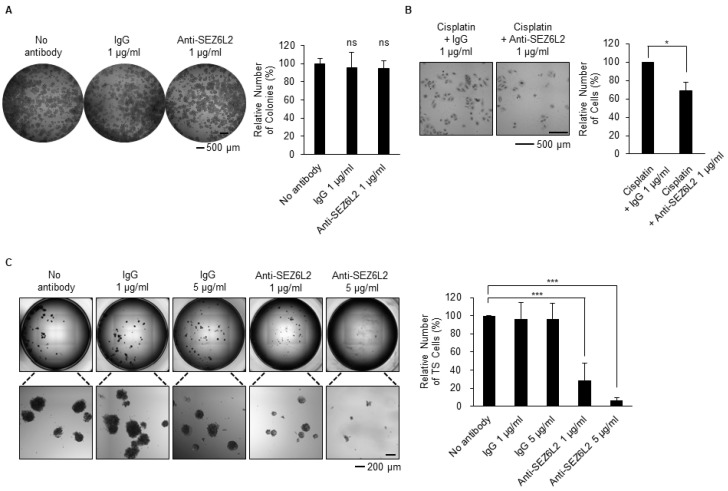
Anti-SEZ6L2 antibody treatment inhibits drug resistance and TS formation. (**A**) H460 cells were seeded at low density and 2D-cultured for 3 days in the presence or absence of an anti-SEZ6L2 antibody (1 μg/mL). Cells that were not treated with antibody and cells treated with the same amount of normal rabbit IgG antibody were used as controls. Whole-cell images from a 96-well plate were obtained using the Cytation 3 cell imaging reader (BioTek). The relative number of colonies (%) was presented as the mean ± standard deviation (n = 4). ns: non-significant to non-antibody-treated cells. (**B**) H460 cells were treated with cisplatin (4 μM) and an anti-SEZ6L2 antibody (1 μg/mL) according to the experimental scheme shown in Figure 1A. Control cells were treated with cisplatin and normal rabbit IgG (1 μg/mL). The relative number of cells (%) was presented as the mean ± standard deviation (n = 3). * *p* < 0.05 relative to normal rabbit IgG antibody-treated cells. (**C**) H460 cells were cultured with TS cells for 7 days in the presence or absence of the indicated concentrations of the anti-SEZ6L2 antibody. Whole-cell images from a 96-well plate were obtained using Cytation 3, and TS cells with a diameter >10 μm were counted. Cells that were not treated with antibodies and cells treated with the same amount of normal rabbit IgG antibody were used as controls. The relative number of TS cells (%) is presented as the mean ± standard deviation (n = 3). *** *p* < 0.001 relative to non-antibody-treated cells.

Next, we aimed to determine whether SEZ6L2 plays a functional role in the acquisition of drug resistance or the formation of TS cells. As SEZ6L2 is a type 1 transmembrane protein with Sushi domains and complement C1r/C1s, Uegf, Bmp1 (CUB) domains in the extracellular portion of the protein [29,30], an anti-SEZ6L2 antibody can be used to neutralize the function of SEZ6L2. First, as a result of the treatment of H460 cells with normal rabbit IgG and anti-SEZ6L2 antibody, it was confirmed that none of them had an effect on cell proliferation in 2D culture (Figure 4A). Next, H460 cells were treated with cisplatin according to the experimental scheme shown in Figure 1A in the presence of an anti-SEZ6L2 antibody or normal rabbit IgG as a control. Enumeration of viable cells showed that treatment with an anti-SEZ6L2 antibody significantly reduced the number of surviving drug-resistant cells (Figure 4B). During TS culture with H460 cells, the addition of the anti-SEZ6L2 antibody significantly reduced TS formation in a dose-dependent manner, whereas the addition of normal rabbit IgG did not (Figure 4C). These results suggest that anti-SEZ6L2 antibody treatment inhibits drug resistance and TS formation.

**Figure 5:**
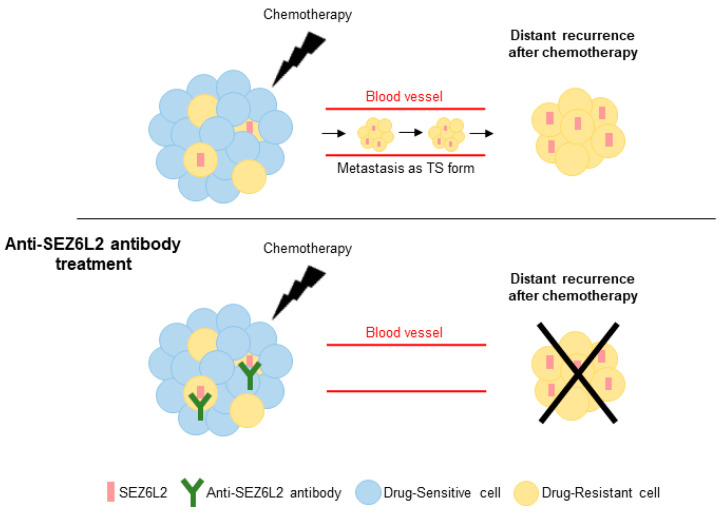
Schematic model of the role of SEZ6L2 in distant recurrence of LUAD after chemotherapy. A small percentage of SEZ6L2-positive cells resided in the primary tumor. The proportion of SEZ6L2-positive cells increased during chemotherapy, and as cancer cells migrated through blood vessels as the suspended state, such as TS cells. Disrupting the role of SEZ6L2 using an anti-SEZ6L2 antibody reduced the numbers of both drug-resistant cells and TS cells.

Overall, these results suggest that SEZ6L2 is upregulated in drug-resistant cells and TS cells and plays functional roles in drug-resistant cell survival and TS formation in LUAD. These results also suggest that drugs targeting SEZ6L2, including antibody therapies, may be used to prevent distant recurrence of LUAD (Figure 5).

## 4. Discussion

Despite great achievements in lung cancer therapy over recent decades, recurrence in distant organs is a major cause of treatment failure, and drug resistance and metastasis contribute to distant recurrence. Therefore, we aimed to define the molecular characteristics of distant recurrence by investigating gene expression changes in drug-resistant cells and TS cells in LUAD. We performed RNA sequencing using drug-resistant cells and TS cells derived from H460 and A549 cells. In the drug-resistant cells, the cell-cycle-related pathway was depressed, and the senescence pathway was activated (Figure 1C), in accordance with previous reports that chemotherapy leads to cell cycle slowdown and cellular senescence [31,32]. Activation of the nucleotide excision repair (NER) pathway is also unsurprising, given its close relationship with drug resistance [28]. The activated glycoprotein VI platelet pathway has no reported relationship with drug resistance, and further studies of this pathway are needed. In TS cells, all activated pathways are related to cholesterol biosynthesis (Figure 2C). This result is in line with the finding that cholesterol biosynthesis is essential for tumorigenesis, cancer stemness, cancer metastasis, and 3D culture of cancer stem cells [33,34,35,36,37], but more research into the role of cholesterol biosynthesis in lung TS cells is needed. Through upstream analysis, we found that *SMARCA4* and *PAF1* are commonly activated upstream regulators in both drug-resistant cells and TS cells (Figure 1C and Figure 2C). SMARCA4 is a transcription regulator in the chromatin remodeling complex, and PAF1 is a component of polymerase associated factor 1 complex, which regulates transcription elongation via RNA polymerase II [38,39]. In previous studies related to lung cancer, both of these regulators have been identified as prognostic markers of NSCLC [40,41]. However, SMARCA4 and PAF1 appear to be activated at the post-transcriptional level, and, therefore, elaborate molecular studies of the mechanisms underlying their activity are required for the development of therapeutics targeting them.

Based on the RNA sequencing data, we found that *SEZ6L2* was commonly upregulated in both drug-resistant cells and TS cells (Figure 3A). The mRNA expression of *SEZ6L2* was also elevated in LUAD patient samples from The Cancer Genome Atlas (TCGA) (Figure 3B). We confirmed that the SEZ6L2 protein was expressed in a small percentage of parental 2D-cultured cells, but this proportion increased during the development of drug-resistant cells or TS formation (Figure 3C,D). As SEZ6L2 is located on the cell surface, its function may be perturbed by an anti-SEZ6L2 antibody. We found that anti-SEZ6L2 antibody treatment reduced the number of cells surviving after drug treatment and TS formation (Figure 4). Recently, antibody therapy has become a predominant class of new drugs used for the treatment of diseases, including cancer, due to the high specificity of these treatments [42,43]. In addition, antibody engineering has advanced dramatically since the first monoclonal antibody was approved by the United States Food and Drug Administration (US FDA) in 1986. For example, Nivolumab, which was the second best-selling monoclonal antibody drug in 2018, targets the PD-1 receptor and has been approved by the US FDA for cancers, including melanoma and NSCLC [43]. Likewise, with improved antibody engineering and further research on SEZ6L2, drug resistance and metastasis could likely be suppressed in LUAD by anti-SEZ6L2 antibody therapy.

It is important to identify the critical factors for CTC survival, as it is essential to survive as CTCs in blood vessels for the primary cancer to spread to other organs. However, there are several limitations in studying the function of CTC in vitro. Currently, ex vivo CTC culture methods derived from patients and patient-derived xenografts have been reported [22,44,45,46], but no in vitro CTC culture methods have been reported. TS culture shows intermediate complexity between 2D in vitro cell culture and 3D in vivo states [13,47] and is similar to CTC culture in that cells grow in a suspended state. In this study, to mimic the characteristics of CTC in vitro, lung cancer cell lines were TS cultured in ex vivo CTC medium. However, since the TS cultured cells in this study are still thought to be close to the previously reported TS than CTC, it is necessary to develop a new method to study CTC in vitro. Nonetheless, the finding that SEZ6L2 is important for survival in suspended culture conditions still suggests that SEZ6L2 will be important in the process of distant cancer metastasis.

In this study, we show a functional role of SEZ6L2 in LUAD, but its detailed molecular mechanism has not been fully explored to date. Some of the functions and mechanisms of SEZ6L2 in cancer and other diseases can be inferred through studies of other proteins in the SEZ6 family. Structurally, all SEZ6 family proteins are transmembrane proteins consisting of Sushi domains and CUB (complement C1r/C1s, Uegf, Bmp1) domains [29,30]. The Sushi domain (also known as complement control protein (CCP) domain or short consensus repeat) and CUB domain in the extracellular regions are related to the complement system, cell adhesion, signal transduction, and protein–protein interactions [48,49,50]. Previous studies of and structural similarities among SEZ6 family members suggest that the extracellular region of SEZ6L2 may be associated with cell-to-cell interactions or signal transduction in LUAD, but further investigation is needed. To conclude, we confirmed that SEZ6L2 was upregulated and played important roles in LUAD drug-resistant cells and TS cells. Our results suggest that anti-SEZ6L2 antibody treatment may be a promising anticancer therapy that reduces the recurrence of lung cancer in distant organs, such as the brain and bones.
